# Orthogeriatric co-management and risk of rehospitalization in older patients with osteoporotic fractures: a retrospective cohort study from Germany

**DOI:** 10.1186/s12877-025-06172-5

**Published:** 2025-07-31

**Authors:** Theresa Unseld, Kilian Rapp, Clemens Becker, Claudia Konnopka, Hans-Helmut König, Andrea Jaensch, Dietrich Rothenbacher, Gisela Büchele

**Affiliations:** 1 Institute of Epidemiology and Medical Biometry, Ulm University, Ulm, Germany; 2 Department of Clinical Gerontology, Robert Bosch Hospital, Stuttgart, Germany; 3 Department of Health Economics and Health Services Research, University Medical Center Hamburg-Eppendorf, Hamburg, Germany; 4 Center for Trauma Research, Ulm University, Ulm, Germany

**Keywords:** Readmission, Geriatric, Rehabilitation, Care home admission, Competing events

## Abstract

**Background:**

As bone mass decreases with age, older people are at an increased risk of fractures, often accompanied by frailty and comorbidities. Specialized geriatric teams can be involved in treating these patients by providing orthogeriatric co-management (OGCM). Previous studies have investigated the effectiveness of OGCM regarding health-related outcomes in older patients with hip fractures. However, evidence regarding adverse health events, such as rehospitalization, and other types of osteoporotic fractures, is limited. This study aimed to investigate the associations between hospital-level OGCM availability and the risk of rehospitalization in patients with osteoporotic fractures.

**Methods:**

This retrospective cohort study was based on health insurance data from 209,885 patients aged $\:\ge\:$80 years who were admitted to a German hospital with one of five types of osteoporotic fractures. We defined patient-relevant states within 180 days after initial hospitalization as discharge to home, transfer to subacute rehabilitation (TSR), institutionalization, rehospitalization, or death, and estimated the hazards of transitions between these states in a multistate model. We defined the control and intervention groups based on the availability of OGCM expertise at the hospital level, which we derived from the annual number of reimbursed procedure codes. We modeled dependencies of the hazards on the time since admission and the time since other post-discharge events.

**Results:**

We found that the association between OGCM availability and the rehospitalization hazard depended on the discharge state and was lowest among patients with TSR. The overall association, estimated across all discharge states, was statistically significant in patients with hip fractures (hazard ratio (HR) and 95% confidence interval 0.91 (0.883, 0.945)) and spinal fractures (HR 0.95 (0.919, 0.992)). There were also statistically non-significant overall reductions among patients with pelvic fractures (HR 0.96 (0.914, 1.005)) or forearm fractures (HR 0.96 (0.915, 1.009)). Among patients with humeral fractures, however, we only observed a reduction in those with TSR (HR 0.88 (0.759, 1.029)) or institutionalization (HR 0.95 (0.880, 1.030)), but not among those discharged to home without TSR.

**Conclusions:**

Our study suggests beneficial associations between OGCM availability and the hazard of rehospitalization, and that the benefit is greatest in combination with subacute rehabilitation.

**Supplementary Information:**

The online version contains supplementary material available at 10.1186/s12877-025-06172-5.

## Background

As bone mass decreases, older people are subject to an increased risk of fracture [1, 2]. In 2019, there were 831,000 incidents of fragility fractures in Europe, which constituted an increase of 22.2 fractures per 1000 individuals compared with 2010 [3]. Adequate treatment of patients with fragility fractures, often accompanied by prevalent comorbidity, is of particular concern due to the implications of early unscheduled hospital readmission (rehospitalization) from individual, health, social, and financial perspectives, and in light of an aging population [4–6]. A comprehensive approach involving multidisciplinary geriatric teams has been initiated as a pivotal strategy to avert functional decline and its associated complications, including rehospitalizations [7–9]. To this end, interdisciplinary geriatric teams have been established, uniting expertise from geriatrics and trauma surgery in the specialized early rehabilitation concept of orthogeriatric co-management (OGCM). In Germany, this concept is formalized in the hospital procedure classification code “OPS8-550” and includes standardized geriatric assessments, frequent interdisciplinary team discussions, medicine tailored to geriatric syndromes, and functionality-focused rehabilitation plans.

Previous evidence suggests that OGCM decreases mortality in patients after hip fracture in Germany and other countries [10–14]. However, evidence is limited regarding the relationship of OGCM with other fracture sites and other outcomes that are relevant for evaluating the clinical effectiveness of OGCM [15–18]. The present study aimed to analyze the association of OGCM with rehospitalization in a nationwide health insurance claims dataset of older patients with index fractures of the hip, pelvis, spine, forearm, or humerus, who were admitted to German hospitals between 2014 and 2019. Conventionally, geriatric research has focused on patients aged 65 and over [19]. The present study focuses on a particularly vulnerable subgroup of patients aged 80 and over, characterized by increased functional decline and comorbidities. Previous claims data studies by our research group have analyzed the associations between the availability of OGCM and patient-relevant outcomes, such as secondary hip fracture, nursing home admission, anti-osteoporotic drug treatment, and mortality in patients with hip fractures [13, 14, 20–22], as well as the association between OGCM and mortality in patients with other fracture types [20]. To evaluate the hazard of secondary hip fracture, they analyzed the relationship between hospital-level rates of OGCM and transfers to subacute rehabilitation (TSR) with secondary hip fractures in patients who underwent surgery for a previous hip fracture [21]. Their results emphasized the importance of time scale and the relevance of post-fracture events to the mechanisms of OGCM. In another claims data study of three German federal states, our research group investigated rehospitalization as a secondary outcome in patients with hip fractures [22]. They found lower rehospitalization rates in the states that primarily offered subacute inpatient rehabilitation compared to a state that primarily offered acute early rehabilitative treatment. Building on previous research, the present study incorporates multistate models to investigate the complex associations of OGCM and rehospitalization, while taking into account post-fracture events such as discharge to home, TSR, admission to nursing home, and death. These associations are analyzed using nationwide claims data from geriatric patients with multiple fragility fractures, thereby expanding the scope beyond commonly studied hip fractures.

## Methods

### Study design, data source, and study population

The association between the availability of OGCM and rehospitalization was analyzed in a retrospective cohort study using claims data provided by the nationwide insurance company “Allgemeine Ortskrankenkasse” (AOK), which is the largest association of health insurance companies in Germany, covering nearly one-third of Germany’s citizens [14]. The anonymized patient-related data were provided by the scientific institute of the AOK (“Wissenschaftliches Institut der AOK”, WIdO). The target population included patients aged $\:\ge\:80$ years who were admitted with an International Code of Diseases (ICD)−10 classified osteoporotic fracture of the hip (more specifically, operated hip fractures, S72.0, S72.1 with reimbursed Operation and Procedure Codes (“Operationen- und Prozedurenschlüssel”, OPS) 5-790, 5-793 or 5-794 in combination with the final digit “e” (femoral neck) or “f” (proximal femur), or OPS 5-820), forearm (S52), humerus (S42), pelvis (S32.1, S32.3, S32.4, S32.5., S32.81, S32.83), or spine (S12.0–S12.2, S12.7, S12.9, S22.0, S22.1, S32.0) between January 1 st, 2014, and December 31 st, 2018 to hospitals in Germany (“index fracture”) [20]. The fractures were classified according to the major discharge diagnoses reported in the claims data. The study population was restricted to patients aged 80 years and over, as the prevalence of frailty and related complications, such as injuries and concurrent diseases, tends to increase with age [19, 23–26]. Therefore, we theorized that older patients would be more amenable to the concepts of tailored geriatric treatment. Patients with implausible death dates, patients with insurance gaps in the time frame of one year before follow-up start to follow-up end, and patients who had experienced the same fracture within 180 days before their index fracture were excluded from the analyses (942 hip fractures (0.9%), 449 spinal fractures (1.1%), 181 pelvic fractures (0.7%), 434 humeral fractures (1.2%), and 142 forearm fractures (0.5%)). The analysis of patients with hip fractures was restricted to those who underwent surgical treatment to exclude outliers, given that surgery is the typical treatment for hip fractures [21], and to minimize the probability that an index fracture constitutes only a follow-up treatment connected to a previously operated fracture. For the other fracture types, patients who had not undergone surgery were also considered in the analysis.

### Dependent variables

The time to rehospitalization per index fracture type (hip, pelvis, spine, forearm, or humerus) was considered the dependent variable of interest. Since patients may die before experiencing a rehospitalization event (and the risk of death is especially high in the older study population), death was considered a competing event. Patients who were still alive and had not been rehospitalized 180 days after their index hospital admission were administratively censored. This censoring was chosen based on previous results suggesting that the index fracture has only a weak influence on later events [21].

### Independent variables

Hospital-level availability of OGCM was defined on an annual basis, contingent upon the presence of at least ten records of the German procedure classification code “OPS8-550” in a given year or the subsequent year. This latter condition, pertaining to the subsequent year, was implemented under the assumption that OGCM concepts might exert an influence on hospital routines prior to their full establishment. The minimum of ten records of OPS8-550 was adapted from a study in stroke research, which assumed hospital-level specialized stroke expertise if a hospital had at least ten billed treatments in stroke units [27]. OGCM can be applied either as joint management in an orthogeriatric unit or as a geriatric liaison service where the patients are first treated in the orthopedic unit, followed by an early transition to a geriatric unit. In both cases, the presence of OPS8-550 coded treatments was considered an indicator of the hospital’s expertise in the field of OGCM. Consequently, a patient’s OGCM level was defined as treatment in a hospital “with OGCM” or “without OGCM”, according to the hospital’s OGCM level in the year of admission. This approach ensured that patients lacking documented evidence of OPS8-550 would still be regarded as beneficiaries of OGCM expertise if the patients were admitted to a hospital currently practicing OGCM (as evidenced by a minimum of ten records of OPS8-550 in the patient’s year of admission or the following year). To mitigate the potential for misclassification, data from hospitals where the transfer rate to another care system was higher than 5% were excluded from the analysis. As baseline covariates, the patient’s sex, age, care level in the month before fracture, and the medication-based comorbidity score [28] (as the number of medication prescriptions for one of 22 predefined disease groups) at hospital admission were considered.

### Statistical analysis

**Fig. 1:**
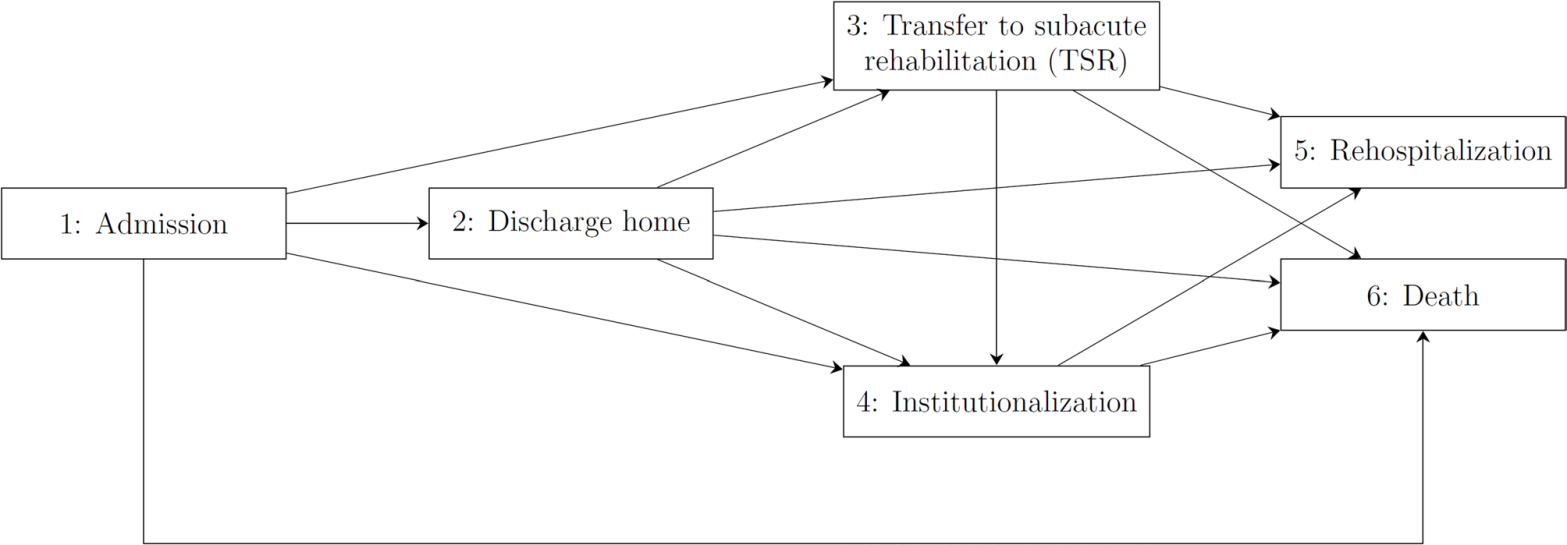
Multistate model describing the possible event-triggered transitions to different patient-relevant states after hospital admission with an osteoporotic fracture of the hip, pelvis, spine, humerus, or forearm.

Baseline characteristics were presented as medians with interquartile ranges or as frequencies with percentages. After admission to the index hospital, patients were considered to be in one of several “states”: admission, discharge to home (short: “discharge”), transfer to subacute rehabilitation (short: “rehabilitation”), institutionalization, rehospitalization, or death **(**Fig. 1**)**. These states were entered upon experiencing post-fracture events, which were either:

- the initial event at which observation starts (hospital admission),
- the event of interest (rehospitalization) or the competing event (death) at which observation stops (“absorbing states”),
- or an intermediate event connected to the discharge state (discharge to home, TSR, institutionalization), which was considered to be associated with the risk for the event of interest (“transient states”).

Throughout the manuscript, when we refer to a “discharge state”, we mean the state that is connected to the locations after discharge. Patients were considered to remain in the state associated with an event until they experienced the next event. Death after rehospitalization was not modeled since rehospitalization was the event of main interest, and only the first rehospitalization was considered in our study. A patient discharged from the hospital to their home will be considered a “discharged home patient” in the “Discharge” state until the time when they are transferred to subacute rehabilitation or institutionalized. From the time of TSR onwards, a patient will be referred to as a “patient with TSR” and their discharge state will be considered TSR (short: “Rehabilitation”), even when they are back home after rehabilitation. No distinction was made between patients who were currently in rehabilitation or those who were previously in rehabilitation and subsequently discharged home, given the small number of patients who were rehospitalized or died after TSR **(Figure ****S1****).** Instead, we assumed that the effects of rehabilitation, whether acute or subacute, extend beyond the therapy phase. Changes in the association of TSR with rehospitalization or death over time (e.g., attenuation of effects as time since TSR increases) were modeled in the non-parametric baseline hazards. Interactions between OGCM and the time since TSR were modeled as covariates in the Cox regression models.

Similarly, once transferred to a nursing home, a patient will be considered an “institutionalized patient” in the “Institutionalization” state, even if transferred back home or to a rehabilitation facility. Transitions to TSR after institutionalization or discharge to home after institutionalization were not modeled since they rarely occurred. To distinguish between patients who completed rehabilitation and returned home and those who completed rehabilitation and were institutionalized, we modeled the former as “patients with TSR” and the latter as “institutionalized patients.” Alternatively, we could have defined a new state, “TSR and institutionalization,” in addition to the state “TSR” (or “at home after TSR”). However, estimating rehospitalization and death hazards was not possible for most fracture types in this alternative model, as there were too few events in these states.

The instantaneous risks of experiencing a rehospitalization event were described by transition hazards and estimated as increments of the Nelson‒Aalen estimator (NAE) or in Cox proportional hazard (PH) models. The NAEs were stratified by the discharge state, OGCM level, and fracture type. The PH models were stratified for the discharge state and fracture type and included OGCM and the other independent variables as covariates. A cluster term was added to account for correlations among patients treated within the same hospital by estimating robust (sandwich) standard errors and confidence intervals [29–31]. The outcome was then defined as the time from discharge, TSR, or institutionalization until rehospitalization, depending on which intermediate event occurred most recently (“clock reset” model). Dependencies of the rehospitalization hazard on the time since index hospital admission and additional intermediate events were addressed by including flexible functions of these times as covariates in the Cox regression models [32, 33].

The association between OGCM and in-hospital death was estimated separately for each level of the length of hospital stay (LoS). As elaborated in further detail in the discussion section, this was done to disentangle the associations between OGCM, LoS, and the rehospitalization hazard. To this end, the level of LoS was defined by the respective 30%, 60%, and 90% quantiles of each OGCM level and fracture type combination (Table S1). All event times were available as daily records, except the time of institutionalization, which was available only as the patient’s institutionalization status at the end of the month. Using the middle of the month as a proxy for the actual institutionalization date. However, this would have introduced immortal time bias for patients who were actually institutionalized later in the month.

The PH assumption of the Cox model was verified by using the Schoenfeld residuals test and graph [34]. For covariates where the PH assumption was in doubt, interactions with time were added. All analyses were performed in R [35]. Details on the estimated multistate model can be found in the Supplement.

## Results

### Description of the study population

The analyzed data included 227,453 index fractures recorded in 209,885 patients admitted to 834 distinct hospitals in Germany (Table 1). The majority of the fractures were fractures of the hip (*N* = 100,245 (44%)), followed by fractures of the spine (*N* = 39,142 (17%)), humerus (*N* = 35,486 (16%)), and forearm (*N* = 28,267 (12%)), and least frequent were fractures of the pelvis (*N* = 24,313 (11%)). Approximately 70% of all fracture types were treated in hospitals where OGCM was available. The analysis of patients with hip fractures was restricted to the 91% who received surgical treatment (for reasons explained in the Methods section). The proportion of surgical treatment for the other fracture types ranged from 5% (pelvis) to 85% (forearm). The baseline characteristics of the unrestricted population, including conservatively treated hip fractures, are presented in Table S3. Most patients were female and did not require long-term care or nursing before their index fracture. Patients with hip or pelvic fractures were, on average, older than patients with spine, humerus, or forearm fractures. Additionally, the median LoS varied by fracture type and was longer in hospitals with OGCM than in those without. Otherwise, the baseline characteristics were similar between patients admitted to hospitals with and without OGCM (Table S2).

**Table 1:** Baseline characteristics by the type of index fracture

**Characteristic**	Index fracture				
	**Hip**, *N* = 100,245^a^	**Pelvis**, *N* = 24,313^a^	**Spine**, *N* = 39,142^a^	**Humerus**, *N* = 35,486^a^	**Forearm**, *N* = 28,267^a^
Orthogeriatric co-management at index hospital	72,376 (72%)	17,287 (71%)	28,643 (73%)	25,312 (71%)	20,039 (71%)
Sex female	78,571 (78.4%)	20,551 (84.5%)	29,839 (76.2%)	30,238 (85.2%)	26,209 (92.7%)
Operated fractures	100,245 (100%)	1,287 (5.3%)	12,594 (32%)	21,924 (62%)	24,035 (85%)
Age at index fracture (years)	87.2 (83.9, 90.7)	87.0 (83.7, 90.5)	85.7 (82.7, 89.0)	85.8 (82.7, 89.3)	85.1 (82.4, 88.5)
Medication-based comorbidity (prescriptions)	4 (3, 5)	4 (3, 6)	5 (3, 6)	4 (3, 6)	4 (3, 5)
Nursing at admission (care level)					
None (0)	35,516 (35%)	9,457 (39%)	18,320 (47%)	17,201 (48%)	16,850 (60%)
Home care (1–2)	18,242 (18%)	5,383 (22%)	8,733 (22%)	6,597 (19%)	4,754 (17%)
Home care (3–5)	21,281 (21%)	4,852 (20%)	6,932 (18%)	6,130 (17%)	3,492 (12%)
Nursing home (1–2)	5,012 (5.0%)	1,125 (4.6%)	1,378 (3.5%)	1,143 (3.2%)	769 (2.7%)
Nursing home (3–5)	20,194 (20%)	3,496 (14%)	3,779 (9.7%)	4,415 (12%)	2,402 (8.5%)
Length of hospital stay in patients discharged alive					
No orthogeriatric co-management at index hospital	13 (11, 16)	8 (6, 13)	8 (5, 14)	9 (5, 13)	5 (3, 7)
Orthogeriatric co-management at index hospital	18 (12, 25)	13 (7, 22)	11 (6, 21)	10 (6, 20)	5 (3, 9)

^a^Median (first, third quartile) or Frequency (%)

### Description of the transition proportions (crude incidences) and stratified hazards

#### Proportions of in-hospital deaths and discharge locations

Most patients were discharged to home at the end of their hospital stays. Fewer than 0.2% were still in the hospital at the end of follow-up, and the proportion of in-hospital deaths was less than 8%, with the highest proportion occurring among patients with hip fractures (Figure S1). Patients with hip fractures were also more often transferred to subacute rehabilitation than patients with other fracture types. For all fracture types, except humeral fractures, the proportion of direct TSR from the hospital was higher than the proportion of TSR after discharge to home. This means that these patients were more often transferred to subacute rehabilitation directly from the hospital than later after discharge. The largest differences in TSR timing were seen in patients with hip fractures (reductions in TSR proportions of 10% with OGCM and 13% without OGCM after discharge to home).

Comparing hospitals with and without OGCM reveals that the former hospitals transferred up to 15% fewer patients to subacute rehabilitation than the latter. The difference in TSR proportions between hospitals with and without OGCM was greatest for patients with hip fractures (22% with OGCM and 37% without OGCM), which aligns with the higher rate of reimbursed inpatient early complex geriatric rehabilitation therapy in terms of OPS8-550 compared to patients with other fracture types.

#### Proportions of rehospitalizations and deaths after discharge

After discharge to home, most patients did not experience any further events. This means that, depending on fracture type and OGCM availability, 23–64% of the discharged home patients remained without TSR, institutionalization, rehospitalization, and death at the end of follow-up. However, patients with hip fractures treated in hospitals without OGCM were rehospitalized slightly more often than they were event-free (29% vs. 23%). Compared to TSR, institutionalization, and death, rehospitalization occurred more frequently (in 29–48% of patients, depending on their discharge state, fracture type, and OGCM level). In patients with TSR and in institutionalized patients, the rehospitalization proportions from hospitals with OGCM were lower than from those without OGCM (greatest reduction among patients with TSR and forearm fractures from 44% without OGCM to 33% with OGCM). Conversely, among patients discharged to home, rehospitalization proportions from hospitals with OGCM were slightly higher than those from hospitals without OGCM (increases ranging from 1% among patients with forearm or spinal fractures to 4% among patients with hip fractures).

When comparing the rehospitalization proportions from different discharge states among patients with the same level of OGCM, a (by up to 9%) higher proportion can be observed in patients who were transferred to subacute rehabilitation from hospitals without OGCM, than in those discharged to home. In patients discharged from hospitals with OGCM, the rehospitalization proportions of patients at different discharge states were more similar (with absolute differences of 3% or less). The proportion of deaths, however, was up to 10% lower in patients with TSR than patients discharged from hospitals to their homes, either with or without OGCM. The only exception to this trend was seen among patients with forearm fractures, for whom the proportion of deaths was 1.2% among those discharged home and 1.6% among those with TSR. Among institutionalized patients, the proportions of both rehospitalizations and deaths were higher than the proportions among patients at the other discharge states, except for patients with pelvic or forearm fractures discharged from hospitals without OGCM (were the proportions were equal or 1% higher in institutionalized patients than in patients with TSR).

#### Causes of rehospitalization

The causes of rehospitalization, categorized by discharge state and fracture type, are presented in the Supplement (Figure S2). The most frequent causes were diseases of the circulatory system (*n* = 15,088; 17% of all rehospitalizations) and secondary injuries of the fracture site (*n* = 14,476; 16%), followed by diseases of the digestive, respiratory, genitourinary, and nervous systems, with percentages ranging from 2.8 to 8%. The top 10 causes also included injuries to another osteoporotic site (*n* = 5,340, 5.8%) or to the head (*n* = 298, 3.3%) and endocrine, nutritional, and metabolic diseases (*n* = 3,608, 3.9%). These frequencies were similar across different fracture types and discharge states. However, some greater differences were found for secondary injuries to the fracture site. These injuries occurred less frequently among patients with TSR than among those discharged home (reductions ranging from 1.8% for spinal fractures to 7.5% for forearm fractures), and more frequently among institutionalized patients (increases ranging from 2% for spinal fractures to 5% for pelvic and humeral fractures compared to patients discharged home).

#### Hazards in different discharge states, rehospitalization, and death in strata of OGCM availability and fracture types

Detailed insights into the transition dynamics leading to the observed event incidences are obtained by considering the NAE curves showing the cumulative hazard of a patient’s transition from one state to another over time (Figure S3). The NAEs clearly reflect that the hazard of rehospitalization was higher than the hazard of death, which aligns with the higher proportion of events for the former event type observed in Figure S1. The course of the NAEs provides information on the timing of events and reveals for each state how fast patients were rehospitalized. For both rehospitalization and death, the post-discharge hazard rates were highest shortly after discharge to either home, TSR, or institutionalization (flattening of the NAE curves).

We were mainly interested in transitions from different states after discharge, since, by definition, patients cannot be rehospitalized while in the hospital. Thus, the only reasons why the hospital admission state is relevant for the estimation and interpretation of the rehospitalization hazard are the following. First, patients may die in hospital, and then not appear in the risk sets for rehospitalization. This can introduce survivorship bias. Second, for patients discharged alive, the in-hospital stay may affect the patients’ constitution at discharge, and thus their discharge state. This can introduce selection bias. As further discussed in the discussion, these types of biases can be addressed by evaluating not only the hazard of rehospitalization, but also the hazard of the competing event death, and by comparing causes for rehospitalization for different discharge states.

It can be seen from Figure S3 that, for all fracture types, patients treated in hospitals with OGCM tended to be discharged slightly later than patients treated in hospitals without OGCM (delayed increase of the NAEs for discharge). Furthermore, the NAEs indicated a lower hazard of in-hospital death with OGCM than without OGCM among patients who were still alive and in the hospital two weeks after the index hospital admission or later. The hazard of discharge to TSR was lower in hospitals with OGCM than without OGCM, whereas OGCM patients were more likely to have already received in-house rehabilitation as part of the OPS8-550 procedure. The difference in the hazards of TSR in hospitals with and without OGCM was more visible for hip, pelvis, or spinal fractures than for humerus or forearm fractures.

### Cox regression models of the hazard of rehospitalization and death

#### Hazard ratios estimated across all discharge states

The estimated hazard ratios (HR) from the Cox regression models (Table 2) quantify the covariate-adjusted differences in the hazard of rehospitalization between hospitals with and without OGCM. The overall HR of rehospitalization estimates the common association of treatment in hospitals with OGCM vs. without OGCM across all discharge states. The greatest benefit of OGCM was found in patients with hip fractures (HR of 0.91 with 95% CI of (0.883, 0.945)), followed by patients with spinal fractures (HR of 0.95 (0.919, 0.992)). In addition, patients with pelvic and forearm fractures had an overall reduced hazard of rehospitalization, but these associations were statistically not significant.

Considering the competing event of “post-discharge death”, the overall hazard was lower in patients with hip, pelvis, and humeral fractures treated in hospitals with than without OGCM. However, statistical significance was only reached for patients with hip fractures. In patients with forearm fractures, the HR was slightly greater than one, though it was estimated with a wide confidence interval due to the small number of deaths within the follow-up time. Among patients with spinal fractures, the overall HR of death was time-dependent, suggesting a protective association at the time of state entry (as time of discharge to home, TSR, or institutionalization) and an increase in the hazard of later follow-up times. The HRs for in-hospital death are presented in Table S4. The estimates indicate protective associations between OGCM and the hazard of in-hospital death, particularly among patients with a hip fracture or a longer LoS.

**Table 2:** Transition-specific hazard ratios for rehospitalization and death in patients treated in hospitals with vs. without orthogeriatric co-management (OGCM) availability

	Fracture type				
	Hip	Pelvis	Spine	Humerus	Forearm
from^b^	HR (95% CI)^a^	HR (95% CI)^a^	HR (95% CI)^a^	HR (95% CI)^a^	HR (95% CI)^a^
to: Rehospitalization					
Discharge	0.93 (0.880,0.983)	1.00 (0.938,1.060)	0.98 (0.934,1.022)	1.03 (0.975,1.085)	0.97 (0.923,1.026)
Rehabilitation	0.89 (0.849,0.931)	0.88 (0.796,0.980)	0.90 (0.820,0.999)	0.88 (0.759,1.029)	0.72 (0.526,0.991)
Institutionalization	0.93 (0.884,0.981)	0.93 (0.846,1.016)	0.92 (0.847,0.990)	0.95 (0.880,1.030)	0.94 (0.846,1.037)
Overall	**0.91 (0.883,0.945)**	0.96 (0.914,1.005)	**0.95 (0.919,0.992)**	1.00 (0.956,1.043)	0.96 (0.915,1.009)
to: Death					
Discharge	1.05 (0.945,1.162)	0.96 (0.821,1.123)	0.88 (0.762,1.021)	0.91 (0.794,1.041)	1.08 (0.841,1.392)
time effect^c^	0.87 (0.810,0.932)	-	-	-	-
Rehabilitation	0.87 (0.707,1.073)	0.73 (0.443,1.214)	-	-	-
Institutionalization	0.83 (0.734,0.948)	0.91 (0.751,1.108)	0.91 (0.683,1.220)	0.76 (0.570,1.016)	1.03 (0.738,1.430)
time effect^c^	1.08 (1.017,1.151)	-	1.16 (1.015,1.332)	1.16 (1.008,1.330)	-
Overall	**0.94 (0.884**,**0.993)**	0.93 (0.830,1.046)	0.86 (0.731,1.009)	0.94 (0.841,1.042)	1.05 (0.587,1.286)
time effect^c^	-	-	1.12 (1.018,1.227)	-	-

^a^Estimated hazard ratios (HRs) with robust 95% confidence intervals (CIs) in clock-reset Cox regression models with baseline hazards stratified by the most recent post-discharge event as the transition origin (“from”). The HRs were adjusted for sex, age, care need, and medication-based comorbidity score at admission, and polynomial functions of the time since index hospital admission and the time since discharge. ^b^HRs were estimated only where at least twenty events were observed in both hospitals with and without OGCM. ^c^Interactions of time (in weeks) since the most recent event with OGCM availability were added where the proportional hazards assumption appeared to be violated based on the Schoenfeld residuals and 95% CIs. Significant overall estimates are highlighted

#### Discharge-location-specific hazard ratios

The discharge-location-specific HRs show the differences in hazards between hospitals with and without OGCM among patients in different discharge states: “Discharge”, “Rehabilitation”, and “Institutionalization”, as described in the section Statistical analysis. When comparing these different discharge states, the most pronounced protective association of OGCM and the rehospitalization hazard was found in patients with TSR, with HRs ranging from 0.72 (0.526, 0.991) for forearm fractures to 0.90 (0.820, 0.999) for spinal fractures. This was followed by institutionalized patients, with HRs ranging from 0.92 (0.847, 0.990) for spinal fractures to 0.94 (0.846, 1.037) for forearm fractures. Among discharged home patients, a protective association of OGCM with the hazard of rehospitalization was observed only for hip fractures (HR 0.93 (0.880, 0.983)), and a non-significant reduction for spinal fractures (HR 0.98 (0.934, 1.022)) and forearm fractures (HR 0.97 (0.923, 1.026)).

The discharge-location-specific HRs for the event “death” confirm the impression of the greater benefit from OGCM in combination with TSR. The smallest HR was observed in patients with TSR and pelvic fractures (0.73 (0.443, 1.214)). However, due to the small number of events (Figure S1), this HR could only be estimated with a very wide confidence interval. For the same reason, no HRs could be estimated for patients with TSR and fractures of the spine, humerus, or forearm.

## Discussion

### Summary of the results

In this nationwide retrospective cohort study, we analyzed 227,453 index fractures of the hip, pelvis, spine, humerus, or forearm in AOK-insured patients aged $\:\ge\:$80 years from Germany who were admitted to the hospital between 2014 and 2019. We found an overall statistically significant reduction in the 180-day hazard of rehospitalization among patients with hip or spinal fractures who were admitted to hospitals with OGCM, compared to those admitted to hospitals without OGCM. Additionally, we found a statistically non-significant reduction in the rehospitalization hazard among patients with pelvic or forearm fractures. Considering differences in the association between OGCM and the rehospitalization hazard between discharge states (discharge to home, TSR, institutionalization), we found that the association was strongest among patients with TSR.

### Comparison to other studies

#### Previous findings on the association of OGCM and rehospitalization

Few previous cohort studies have investigated the associations between OGCM and the hazard of rehospitalization [36–40]. A register study of patients with hip fractures from Sweden found a protective association of OGCM with the hazard of rehospitalization within 30 days (covariate-adjusted HR of 0.79 (0.71, 0.88)) [37]. A cohort study conducted in a Chinese medical department found a statistically non-significant reduction in the 28-day overall rehospitalization rate from 14.9% before the implementation of OGCM to 12.6% afterward [38]. The Chinese study reported that the reduction in the rehospitalization rate resulted mainly from a reduction in readmissions due to medical reasons, whereas the rehospitalizations due to orthopedic reasons increased. In contrast, a cohort study of hospital discharge records from hospitals in the Bologna Metropolitan Area in Italy reported a smaller (but non-significant) odds ratio of hospital rehabilitation programs vs. no rehabilitation for orthopedic rehospitalizations than for all-cause rehospitalization [36].

The non-significance of the results in the Chinese and Italian studies, and the lower HR in the Swedish study compared to our study, might be a consequence of differences in the OGCM program in Germany, China, and Sweden. For example, the median LoS for patients with hip fractures was 8 days with OGCM and 7 days without OGCM in the Chinese study, 15 days with OGCM and 13 days without OGCM in the Swedish study, and 18 days with OGCM and 13 days without OGCM in our study. This allowed for a longer OGCM application period in the Swedish and our studies than in the Chinese study. Another difference between the Chinese and Italian studies and our study was the lower sample size and the associated lower statistical power in the former studies. Additionally, the Swedish and Chinese studies had shorter follow-up periods and on average younger patients with hip fractures (82 years in the Swedish patients, 84 years in the Chinese and Italian patients vs. 87 years in our study’s patients), and age is a significant predictor of rehospitalization [39].

#### Time-dependent associations and associations with length of stay

Moreover, all of the cited previous studies varied in their investigated baseline covariates and statistical methods. The Swedish study reported a lower HR for rehospitalization at the beginning of follow-up than at later times and used logistic regression models to address the associated violations of the PH assumption [37]. In our study, we found that the hazard of rehospitalization was highest directly after discharge. We addressed this trend by modeling the dependencies of the hazards on multiple time scales, such as time since hospital admission, and time since discharge, TSR, or institutionalization. Then, we addressed the remaining violations of the PH assumption by adding interactions of the covariates with the follow-up time. The non-proportional hazards observed in the Swedish study may be explained by the associations between the patients’ LoS and both OGCM and the rehospitalization hazard. At the beginning of the follow-up period, patients who had already been discharged from hospitals with OGCM must have had a similar LoS than patients who had already been discharged from hospitals without OGCM. However, at later follow-up times, variability in LoS among discharged patients can increase due to prolonged OGCM treatment. This modifies the association between OGCM and the hazard of rehospitalization through its association with LoS at later follow-up times.

Insights from a multistate model of surgical patients indicate that LoS is statistically significantly associated with both the hazard of 30-day rehospitalization and death [41]. As we observed in our study, the hazards of rehospitalization and death in that multistate model were highest immediately after discharge and decreased thereafter. Furthermore, the aforementioned study reported a statistically significant association of prolonged LoS with poorer preoperative health status and greater patient age. In our present study, however, the situation was more complicated. LoS could be used neither as a proxy confounder for patients’ preoperative health nor as an indicator of treatment success. This is because, due to the nature of the data, we could not determine whether prolonged LoS is a sign of poorer preadmission health or the result of enhanced orthogeriatric care with OGCM. Specifically, OGCM treatment, as defined by procedure code OPS8-550, formally requires a LoS of at least seven days for in-hospital early geriatric rehabilitation to be completed [13, 14]. A meta-analysis by Grigoryan et al. [42] of patients with hip fractures reported a reduced hazard of in-hospital death and shorter LoS with geriatric interventions on orthopedic wards. In the context of our study, however, a longer-than-average LoS does not necessarily imply a more complicated fracture or a poorer health condition that prevents early discharge. Nevertheless, even after adjusting for the OGCM availability and the prognostic baseline covariates, we observed statistically significant associations between LoS and the hazards of rehospitalization and post-discharge death. These observations suggest that the association between LoS and the hazard of rehospitalization or death is not fully explained by the hospital-level OGCM availability or baseline covariates.

#### Discharge-destination- and outcome-specific associations between OGCM and additional predictors for the rehospitalization hazard

Besides patients’ age and LoS, the time to surgery, dementia, renal function, systolic blood pressure, arrhythmia, and comorbidity have been reported as key predictors for rehospitalization [39, 41, 43]. In our study, we found significant associations between the Huber comorbidity score and the rehospitalization hazard of all fracture types and discharge states. This indicates that comorbidity remains an important predictor of rehospitalization, even when adjusting for OGCM. However, we also found that the distribution of Huber comorbidity scores was very similar among patients with different fracture types who were admitted to hospitals with and without OGCM. The similarity of comorbidities in a geriatric population limits the usefulness of comorbidities as a predictive factor and is the reason why other authors have suggested focusing efforts on modifiable risk factors instead, such as time to surgery or delirium [39].

A multicenter cohort study of patients discharged from acute geriatric units in France found that some characteristics, such as weight loss during the initial hospital stay, or loss of independence, are only associated with one of the hazards of either rehospitalization or death, but not both [44]. Moreover, the study reported that institutionalization increases the hazard of death, but decreases the hazard of rehospitalization. The Swedish study confirmed these discharge-destination-specific associations between OGCM and the hazard of death [37]. More specifically, they found a 13% increase in 30-day mortality for each day reduction in LoS among patients with a LoS $\:\le\:$10 days discharged to short-term nursing homes. The authors hypothesize that this may be a consequence of particularly vulnerable patients being discharged too early to nursing home homes, before they can receive proper treatment, including medical evaluation, recovery, and rehabilitation. However, the Swedish study did not investigate such discharge-destination-specific associations with the hazard of rehospitalization.

In our study’s multistate model, we examined how the associations between OGCM and transition hazards depend on both the final event of interest (rehospitalization or death) and patients’ discharge states. We found both higher proportions and hazards of rehospitalization in patients treated in hospitals without OGCM than those treated in hospitals with OGCM. However, our study could not confirm the previously reported reduction in rehospitalization proportions for institutionalized patients compared to discharged home patients, nor could it confirm statistically significant discharge-location-specific associations of LoS and the hazard of death [37, 41]. This difference may be due to the less granular distinction of discharge locations in our study or the imprecise (monthly) reporting of institutionalization. Additionally, while we examined the association between LoS and the hazard of in-hospital death, we excluded LoS as a predictor of the rehospitalization hazard and post-discharge hazards, since our estimand was designed to be a combination of the direct and indirect (via LoS) associations of OGCM with these hazards.

### Discussion of the results

#### Mechanisms by which OGCM might reduce the rehospitalization hazard

The reasons for the observed associations are speculative. The standardized and well-structured complex early geriatric rehabilitation (OPS8-550), which includes 20 therapeutic sessions - comprising for example physiotherapy, occupational therapy, speech therapy (if indicated), and social services - within a 14-day period, may improve physical capacity [45]. Additionally, OGCM is designed to reduce perioperative complications through early intervention plans, targeted management of existing comorbidities, close monitoring, and coordinated postoperative care by a multidisciplinary team of geriatricians, anesthetists, intensivists, surgeons, physicians, nurses, therapists, and general practitioners [46, 47]. Secondary injuries to the primary fracture site, injuries to another osteoporotic site, and disorders of bone density and structure accounted together for 21,385 cases, which is almost one-quarter of all rehospitalizations in our study. Early geriatric rehabilitation in hospitals with OGCM may reduce the hazard of rehospitalizations related to secondary fractures or functional decline [21]. Better timing of surgery [48, 49] and better management of comorbidities and anti-osteoporotic drug treatment [21, 50] can contribute to this benefit in hospitals with OGCM.

Another major cause of rehospitalization was diseases of the circulatory system. In a previous investigation of heart failure-related rehospitalizations, patients reported inappropriate fluid balance, premature hospital discharge, and deficits in ambulatory follow-up care [51]. OPS8-550 standards are designed to address these factors. This becomes evident, for example, from the longer average LoS in hospitals with OGCM allowing for more intensive perioperative care and monitoring. These standards may also reduce complications related to the digestive, genitourinary, and respiratory systems, which we reported as further top-10 causes of rehospitalization. Finally, OPS8-550 standards also ensure the appropriate provision of assistive devices and facilitate high-quality discharge management. This includes effective communication with family members and the organization of domestic support services or outpatient nursing care. The combined effect of these coordinated interventions likely explains the observed associations.

#### Differences in the OGCM associations between fracture types and discharge States

Early complex geriatric rehabilitation is most commonly used for patients with hip fractures. This is reflected in the higher proportion of OPS8-550 reimbursements (34% for hip fractures vs. 26% for pelvic fractures, 21% for spinal fractures, 18% for humerus fractures, and 7% for forearm fractures) and may explain why the strongest association was observed in this patient subgroup. Meanwhile, the non-significance of the overall HRs in patients with pelvic or forearm fractures may be due to the slightly greater point estimates, the smaller sample sizes for these fracture types compared to hip or spinal fractures, or due to a greater diversity in the treatment for these fracture types, as reflected in the wider CIs. The neutral overall HR among patients with humeral fractures resulted from the contrasting associations in patients discharged to home vs. in institutionalized or non-institutionalized patients with TSR. While the HRs in the latter discharge states were similar between humeral and other fracture types, the HR in the discharged home state was slightly greater than one.

#### LoS in hospitals with OGCM and death as competing risk

The OGCM-associated reduction in the hazard of rehospitalization did not come at the cost of an increased hazard of death. Except for a beneficial association among patients with hip fractures, the HRs for post-discharge death were statistically non-significant when estimated across discharge states. Furthermore, a protective association between OGCM and the hazard of in-hospital death was observed among patients with relatively long LoS for a given fracture type or OGCM level. Among patients discharged home, a prolonged LoS is usually considered to be associated with higher frailty and more comorbidities [37, 41]. To disentangle the associations between LoS and the hazard of in-hospital death from the associations between LoS and OGCM and the fracture type, we reported the HRs separately for OGCM- and fracture-type-specific strata of LoS. We consider the main reason why patients in hospitals with OGCM had a longer average LoS than patients without OGCM to be the early rehabilitation program in hospitals with OGCM [13].

Among institutionalized patients with hip, spine, or humeral fractures, we observed an initial protective association of OGCM with the hazard of post-discharge, followed by a significant increase in the hazard at later follow-up times. This may be due to survivorship bias, whereby vulnerable patients discharged from hospitals without OGCM may die sooner after institutionalization than those discharged from hospitals with OGCM. Furthermore, the finding of a lower HR of both rehospitalization and death in institutionalized patients than in patients discharged home suggests that the issue of premature discharge to nursing homes, which was observed in the Swedish study [37], could be properly addressed by the concepts of OGCM.

#### Implications of the findings for healthcare policy and practice

Prolonging the time from hospital discharge to rehospitalization benefits both patients and the healthcare system. Patients can spend more time outside the hospital, and hospitals can allocate more resources to other patients. This is especially relevant given an aging population and evidence suggesting a generally longer LoS, higher rates of subsequent rehospitalization, and higher mortality rates among rehospitalized patients [39, 44, 52]. The observed associations suggest that patients should be transferred more frequently to subacute rehabilitation or nursing homes than to their homes and that they should be transferred directly to subacute rehabilitation after discharge to maximize the overall benefit of OGCM. Although the OPS8-550 concept includes in-hospital rehabilitation, transferring patients to rehabilitative facilities or nursing homes may be necessary to preserve the geriatric achievements due to these facilities’ superior resources, such as persistent encouragement for functional recovery and better assistance with daily activities. While it may not be feasible to transfer every patient to subacute rehabilitation, healthcare providers should strive to promote accessibility to these facilities.

### Strengths and limitations

#### Claims data and study design

Our study has several strengths and limitations. First, the use of health claims data allowed us to include a large number of patients and hospitals in our study, thereby increasing the information content of the results. Second, we had no response bias, as all available patients within this claims database could be included in the statistical analysis. However, due to the nature of the claims data, we could not adjust for other potentially confounding covariates, such as lifestyle, complications, social support, or detailed functional status. In particular, our previous study showed that patients treated in hospitals with lower rates of OGCM typically receive poorer anti-osteoporotic drug treatment than patients treated in hospitals with high rates of OGCM [21]. Thus, anti-osteoporotic drug treatment may have mediated parts of the observed associations between OGCM and the hazard of rehospitalization. Third, we excluded hospitals without OGCM that transferred more than 5% of their patients to hospitals with OGCM, and vice versa. Although these hospitals would have provided additional data, excluding them avoids both exposure misclassification and selection bias.

On the other hand, the use of health claims data implied limitations regarding the accuracy of the timing of events. One limitation is that the patients’ institutionalization statuses were only recorded monthly at the end of each month. As a consequence, patients who were institutionalized at any time before the last day of the respective month were still considered to be in the previous discharge state. For patients who were already institutionalized before their fracture, the time of institutionalization was set equal to the time of hospital discharge. However, since this imprecise ascertainment was applied regardless of the OGCM availability, it is not expected to systematically bias the association with rehospitalization or death. A second limitation is the delay in recording between the actual fracture and hospital admission. This is particularly true for spinal fractures [53], where the trauma is often small and the onset of pain may be slower than for other fracture types. This makes it difficult to determine the date of the fracture, which increases the variability of the time between the fracture and hospital admission. While this may lead to inaccurate estimations, it is not expected to bias our results systematically, as this limitation relates to the type of fracture rather than the type of hospital treatment.

#### Evaluation of orthogeriatric co-management

In our study, we examined the association between OGCM and rehospitalization at the hospital level rather than at the individual patient level. This approach was adopted based on our previous studies [14, 20], to mitigate exposure misclassification, selection bias, and immortal time bias. More specifically, defining OGCM based on patients’ individual records of reimbursed OPS8-550 would introduce immortal time bias in an analysis that evaluates OGCM availability from the time since follow-up start, since patients must be discharged alive to have reimbursed OPS8-550. An analysis that compares only discharged patients would circumvent immortal time bias, but it would still not account for selection bias, since OPS8-550 may not be feasible for some patients due to their functional condition. In addition, exposure misclassification could occur in patients who received the major components of OPS8-550, but lacked some minor components, and were therefore not eligible for reimbursement. The non-significant association of the OGCM availability with the hazard of death in patients discharged home could then be a consequence of not only unmeasured confounders, or the small number of events, but also of the small proportion of actually reimbursed OPS8-550 in hospitals with OGCM availability. These small proportions may have provided a treatment signal that was too weak to detect. This problem could have been avoided with an intention-to-treat analysis. However, such an analysis was not possible due to the nature of the claims data. In addition, defining OGCM based on actual reimbursed OPS8-550 would fail to reflect the expertise and routines of OGCM that are available and could benefit all patients at the hospital level.

#### Multistate model, time scales, and statistical analysis

We used a multistate model with multiple time scales to account for the complex temporal dynamics and intermediate events, thereby enhancing the accuracy of risk estimation. For example, a patient must first survive the initial hospitalization, then be discharged to home, to a rehabilitation facility, or a nursing home, and survive before ultimately being rehospitalized. As previous studies have shown [37, 41], both the discharge time and location may affect the hazards of death or rehospitalization. In a hospital or nursing home, vulnerable patients can be provided with better resources than at home. To examine whether such differences in discharge affect the hazard of rehospitalization, and to rule out the possibility that a reduction in the probability of rehospitalization comes at the cost of higher mortality, we evaluated the hazard of rehospitalization in different discharge states and evaluated death as a competing risk. Because the timing of discharge may depend on patient frailty [41], we defined relative levels of LoS as the 30%, 60%, and 90% quantiles of each combination of OGCM level and fracture type. Within each LoS level, we then estimated stratified HRs for in-hospital death to mitigate the possibility that the higher hazards in hospitals without OGCM were due to a different LoS-associated frailty constitution of the non-discharged patient population than in hospitals with OGCM.

Furthermore, the inclusion of multiple time scales allowed for associations of the hazard of rehospitalization with not only the time since hospital admission but also with the time since discharge to home and the time since TSR or institutionalization. We note, however, that the sample size becomes quite small at follow-up times of 150 days or later for some of the latter timescales. This is because only those individuals who were admitted to the index hospital no more than 180 days ago, who have not been rehospitalized, and who have not died are considered to be at risk of rehospitalization at a given follow-up time. However, the confidence intervals of the overall OGCM estimates for the hazards of rehospitalization and death were not wider than twice the hazard reduction, and the regression coefficients were estimated over the entire period from discharge to home, TSR, or institutionalization to rehospitalization, death, or censoring. Therefore, we consider the estimation to be valid and to reflect the main trends. In addition, we used robust sandwich estimators for the variances in the Cox models to account for correlations of patients treated within the same hospital [29–31]. To test the PH assumption and to relax it when in doubt, we examined interactions of OGCM and baseline covariates with follow-up time. We expect that these measures in the statistical analysis will further strengthen the validity of our results.

#### Generalizability of the results

Using nationwide claims data from one, (although the largest) German health insurance company may limit the generalizability of our results. In particular, the observed results may not be generalizable to older people insured by different insurance companies or older people living in different countries. For instance, Germany’s policy of providing healthcare regardless of a citizen’s financial status may have resulted in different mortality and rehospitalization rates than what would have been observed in a country without such a policy. However, due to the large insurance coverage of this company (approximately 27 million insured persons in the last year of the study’s inclusion period 2018 [54]), we anticipate that our findings will be representative of the majority of Germany’s mandatory insurance population. Additionally, the encouragement of acute geriatric care in other countries (e.g [55])., extends the relevance of our study’s results.

Finally, the results observed in the inpatient cohort with forearm fractures and a median LoS of five days may not be generalizable to other patients with forearm fractures. While other studies have reported higher proportions of outpatient cases [56, 57], the admission of an inpatient patient may indicate increased frailty or related conditions. This shifts the focus of the generalizability of the results observed in the present cohort from patients with this particular fracture type to frailty patients in general.

## Conclusion

In conclusion, we found that treatment in hospitals with the availability of OGCM was statistically significantly associated with an overall reduced hazard of rehospitalization in older patients with osteoporotic fractures of the hip or spine. The overall hazard of the competing event “death before rehospitalization” was statistically significantly reduced in patients with hip fractures. The association of OGCM availability with the hazards of rehospitalization or death varied depending on whether the patients were discharged home, transferred to subacute rehabilitation, or institutionalized. For all fracture types, the greatest benefit of OGCM was seen in patients with TSR.

The present study extends the existing literature on the association of OGCM with patient-relevant outcomes and the analysis of post-fracture events. It addressed the association of hospital-level OGCM availability with the hazard of rehospitalization in a multistate model considering patient-relevant events after hospital admission. Results were based on a large set of nationwide health claims data, including not only patients with hip fractures but also other fracture types. The observed statistically significant reduction in the hazard of rehospitalization in patients with hip or spinal fractures highlights the importance of geriatric care systems and is of particular interest in light of an aging population.

## Supplementary Information

Supplementary Material 1: Supplementary Material 1: Supplement (Supplement.pdf): “Supporting information for orthogeriatric co-management and risk of rehospitalization in older patients with osteoporotic fractures: a retrospective cohort study from Germany” providing details on the statistical analysis and supplementary tables (Tables S1, S2, S3, S4) and figures (Figures S1, S2, S3, S4).

## Data Availability

The data analyzed during the current study cannot be shared because they originated from the WIdO and the use is contractually bound.

## Abbreviations

AOK: Allgemeine Ortskrankenkasse
CCI: Charlson comorbidity index
HR: Hazard ratio
LoS: Length of hospital stay
NAE: Nelson-Aalen estimator
OGCM: Orthogeriatric co-management
OPS: Operation and procedure codes
PH: Proportional hazards
TSR: Transfer to subacute rehabilitation
WIdO: Scientific institute of the AOK
