# Supplementary material for: Orthogeriatric co-management and risk of rehospitalization in older patients with osteoporotic fractures: a retrospective cohort study from Germany
Source: BMC Geriatr. 2025 Jul 31;25:566. doi: 10.1186/s12877-025-06172-5 (PMC12312450; doi:10.1186/s12877-025-06172-5)
Supplement: Supplementary file 1 — Supplementary Material 1: Supplementary Material 1: Supplement (Supplement.pdf): “Supporting information for orthogeriatric co-management and risk of rehospitalization in older patients with osteoporotic fractures: a retrospective cohort study from Germany” providing details on the statistical analysis and supplementary tables (Tables S1, S2, S3, S4) and figures (Figures S1, S2, S3, S4). [file 12877_2025_6172_MOESM1_ESM.pdf]

Supporting information for
Orthogeriatric co-management and risk of rehospitalization in older patients with osteoporotic fractures: a retrospective cohort study from Germany

Details on the statistical analysis

The possible transitions between the selected patient-relevant states after hospital admission (Figure 1, main manuscript) were described by a time process $X(t)$ for times t until the end of follow-up (180 days). Individuals were considered to remain in the state corresponding to their most recent event (hospital admission, discharge to home, transfer to subacute rehabilitation (TSR), institutionalization, rehospitalization, or death) until they experienced the next event. For example, patients with no further event after hospital admission were considered to be in the "admission" state at the end of follow-up, and patients without any further event after discharge to home were considered to be in the "discharge" state at the end of follow-up (or at the time of censoring). The instantaneous risks of moving from one state $g \in \{1,2,3,4\}$ (admission, discharge to home, TSR, or institutionalization) to another state $h \in \{2,3,4,5,6\}$ (discharge to home, TSR, institutionalization, rehospitalization, or death), or remaining in the same state g, were defined as transition hazards:

$$\alpha_{gh}(t) = \lim_{\Delta t \to 0} \frac{1}{\Delta t} P(X(t+\Delta t) = h | X(t) = g, \mathcal{F}_{t^-})$$
$$\overset{\text{Markov}}{=} \lim_{\Delta t \to 0} \frac{1}{\Delta t} P(X(t+\Delta t) = h | X(t) = g), \qquad g \neq h$$
$$\alpha_{gg}(t) = -\sum_{h, h\neq g} \alpha_{gh}(t)\,,$$

where the sigma-algebra $\mathcal{F}_{t^-} = \sigma((X(s)), s < t)$ describes the history of transitions at time t^-, consisting of the visited states and transition times before time t. For the basic time scale, a "clock-reset" approach was considered where the time t refers to the time since entry into the current state, i.e. the time of the most recent event.

Of primary interest were the associations of the hospital-level orthogeriatric co-management (OGCM) availability,

$$\mathrm{Z_{OGCM}} = \mathbb{1}(\text{index treatment in a hospital where OGCM was available})$$

with the transition hazards for rehospitalization or the competing event post-discharge death. These associations were quantified in Cox regression models

$$\alpha_{gh}(t|\mathrm{Z_{OGCM}}, Z, d) = \alpha_{gh;0}(t)\exp(\delta_{gh}\mathrm{Z_{OGCM}} + \beta_{gh}^T Z + \gamma_{gh}(d_1)\mathbb{1}(\mathrm{t} > \mathrm{d_1}) + \zeta_{gh}(d_2)\mathbb{1}(\mathrm{t} > \mathrm{d_2})), \tag{1}$$

where $\alpha_{gh;0}(t)$ represents the nonparametric baseline hazard of $g \to h$ transitions for $g \in \{2,3,4\}, h \in \{5,6\}$, Z the vector of baseline covariates, and

$$\gamma_{gh}(d_1) = \sum_{k=0}^{2} \gamma_{ghk} \cdot d_1^k,$$
$$\zeta_{gh}(d_2) = \sum_{k=0}^{2} \zeta_{ghk} \cdot d_2^k,$$

are polynomial functions of the times $d = (d_1, d_2)$, as the times since index hospital admission, or discharge. These polynomial functions model the dependencies on multiple time scales for times when state entry occurred after hospital admission or discharge [1]. The functions were evaluated for a discrete time grid of follow-up weeks, starting with weekly intervals in the follow-up weeks one to twelve weeks after admission, and continuing with monthly intervals. For transitions where d_1 or d_2 was equal to or less than the time since state entry, t, their value was set to zero and their association with the corresponding transition hazard was not estimated. The hospital length of stay (LoS) was not included as a covariate in model (1) because interest lay in an estimation of the association of OGCM with rehospitalization, irrespective of

the LoS, and the LoS was considered a mediator of the causal path OGCM→ LoS → rehospitalization/death. This is because the time of discharge to either home, subacute rehabilitation, or nursing home depends on the level of OGCM, and the LoS also affects the time of rehospitalization and death.

The regression coefficients (log hazard ratios (HR)) of the baseline covariates, $\gamma_{gh}^T = (\gamma_{gh0},\ \gamma_{gh1}, \gamma_{gh2})$, the time functions, $\zeta_{gh}^T = (\zeta_{gh0},\ \zeta_{gh1}, \zeta_{gh2})$, and the OGCM availability, δ_{gh} were estimated by the partial maximum likelihood method, while the baseline hazards, $\alpha_{gh;0}(t,)$ were estimated by the Breslow estimator. Besides the transition-specific OGCM coefficients, "overall OGCM" effects, $\delta_h, h = 5{,}6$, were estimated for the hazards for rehospitalization or post-discharge death by using a joint model of all transitions to these states. This joint model was implemented with stratified baseline hazards for the transient states (discharge, TSR, institutionalization) and under the assumption of proportionality of the baseline hazards of these transient states with respect to the regression coefficients [2].

For the hazard of in-hospital death, the LoS was divided into four intervals defined by the 30%, 60%, and 90% quantiles of each combination of fracture type and level of OGCM at the index hospital **(Table S1).** For each LoS level, the OGCM HR for in-hospital death was then estimated in a model of the time t_s spent in that LoS level and with LoS-stratified baseline hazards $\alpha_{16;0s}(t_s)$:

$$\alpha_{16;s}(t_s|\mathrm{Z_{OGCM}}, Z) = \alpha_{16;0;s}(t_s)\exp\left(\delta_{16;s}\mathrm{Z_{OGCM}} + \beta_{16}^T Z\right)$$

This model allowed the association of OGCM with the hazard of in-hospital death to vary depending on the LoS. The advantage of this model over a model where time was defined as time since admission is that it keeps the risk sets of hospitals with and without OGCM comparable for each time during the index hospital stay. Otherwise, the association of OGCM could be biased because LoS is both a proxy for a patient's pre-admission health status [3] and a consequence of the prolonged duration of acute rehabilitative treatment as defined by the procedure code OPS8-550 in OGCM hospitals. More specifically, at later follow-up times (e.g., 6 to 20 days after discharge), patients with a LoS classified as "short" or "regular" in hospitals without an OGCM had already been discharged, while those with a "long" or "prolonged" LoS remained. In contrast, the risk set in hospitals with OGCM at those times consisted of patients with a long or prolonged LoS, as well as those with a typical or regular LoS for early complex geriatric rehabilitation **(Table S1).** Since LoS is associated with patients' frailty and functional status [3, 4], estimating the hazard ratio for in-hospital death in hospitals with versus without OGCM without considering the association with LoS could lead to selection bias and overestimation of the OGCM association with the hazard of in-hospital death.

Supplementary Tables and Figures

Table S1: Classification of length of hospital stay (LoS) according to the 30%, 60%, and 90% quantiles of each combination of fracture type and level of orthogeriatric co-management (OGCM) at the index hospital.

Index fracture	OGCM	short LoS	regular LoS	long LoS	prolonged LoS
Hip	no	[0,11)	(11,14]	(14,22]	(22,180]
Hip	yes	[0,12)	(12,20]	(20,33]	(33,180]
Pelvis	no	[0,6)	(6,10]	(10,20]	(20,180]
Pelvis	yes	[0,7)	(7,17]	(17,29]	(29,180]
Spine	no	[0,6)	(6,10]	(10,21]	(21,180]
Spine	yes	[0,7)	(7,15]	(15,29]	(29,180]
Humerus	no	[0,6)	(6,10]	(10,18]	(18,180]
Humerus	yes	[0,7)	(7,13]	(13,28]	(28,180]
Forearm	no	[0,3)	(3,5]	(5,11]	(11,180]
Forearm	yes	[0,3)	(3,6]	(6,19]	(19,180]

Table S2: Baseline characteristics of patients grouped by the level of orthogeriatric co-management provided at the index hospital.

	**Orthogeriatric co-management at the index hospital**	
**Characteristic**	**no**, N = 63,796[1]	**yes**, N = 163,657[1]
Sex female	52,183 (81.8%)	133,225 (81.4%)
Operated fractures	44,817 (70%)	115,268 (70%)
Age at index fracture (years)	86.4 (83.2, 89.9)	86.4 (83.2, 90.0)
Medication-based comorbidity (prescriptions)	4 (3, 6)	4 (3, 6)
Nursing at admission (care level)		
None (0)	28,429 (45%)	68,915 (42%)
Home care (1-2)	11,624 (18%)	32,085 (20%)
Home care (3-5)	11,751 (18%)	30,936 (19%)
Nursing home (1-2)	2,623 (4.1%)	6,804 (4.2%)
Nursing home (3-5)	9,369 (15%)	24,917 (15%)

[1]Median (first, third quartile) or Frequency (%)

Table S3: Baseline characteristics by the type of index fracture, including non-operated hip fractures.

	**Index fracture**				
**Characteristic**	**Hip**, N = 109,931[1]	**Pelvis**, N = 24,386[1]	**Spine**, N = 39,508[1]	**Humerus**, N = 35,869[1]	**Forearm**, N = 28,355[1]
OGCM at index hospital	79,942 (73%)	17,391 (71%)	29,003 (73%)	25,753 (72%)	20,184 (71%)
Sex female	85,932 (78.2%)	20,607 (84.5%)	30,109 (76.2%)	30,571 (85.2%)	26,286 (92.7%)
Operated fractures	100,439 (91%)	1,295 (5.3%)	12,735 (32%)	21,961 (61%)	24,042 (85%)
Reimbursed OPS8-550	36,948 (34%)	6,492 (27%)	8,214 (21%)	6,643 (19%)	2,087 (7.4%)
Age at index fracture (years)	87.2 (83.8, 90.7)	87.0 (83.7, 90.5)	85.7 (82.7, 89.0)	85.8 (82.7, 89.3)	85.1 (82.4, 88.5)
Medication-based comorbidity (prescriptions)	4 (3, 6)	4 (3, 6)	5 (3, 6)	4 (3, 6)	4 (3, 5)
Nursing at admission (care level)					
No nursing (0)	38,717 (35%)	9,458 (39%)	18,474 (47%)	17,374 (48%)	16,877 (60%)
Home care (1-2)	20,288 (18%)	5,412 (22%)	8,841 (22%)	6,702 (19%)	4,793 (17%)
Home care (3-5)	23,441 (21%)	4,887 (20%)	7,026 (18%)	6,203 (17%)	3,499 (12%)
Nursing home (1-2)	5,479 (5.0%)	1,132 (4.6%)	1,379 (3.5%)	1,154 (3.2%)	768 (2.7%)
Nursing home (3-5)	22,006 (20%)	3,497 (14%)	3,788 (9.6%)	4,436 (12%)	2,418 (8.5%)
Length of hospital stay in patients discharged alive					
no OGCM	13 (10, 16)	9 (6, 13)	8 (5, 13)	9 (5, 13)	5 (3, 7)
OGCM	18 (11, 25)	13 (7, 22)	11 (6, 21)	10 (6, 20)	5 (3, 9)

[1]Median (IQR) or Frequency (%)

OGCM: orthogeriatric co-management

Table S4: Transition-specific hazard ratios for in-hospital death in patients treated in hospitals with vs. without orthogeriatric co-management (OGCM) availability.

	Fracture type				
	Hip	Pelvis	Spine	Humerus	Forearm
**from†**	**HR (95% CI)***	**HR (95% CI)***	**HR (95% CI)***	**HR (95% CI)***	**HR (95% CI)***
**to: Death**					
Admission, short LoS	0.92 (0.855,0.998)	0.93 (0.733,1.192)	1.23 (0.974,1.551)	0.90 (0.712,1.127)	-
Admission, regular LoS	0.80 (0.681,0.929)	0.94 (0.680,1.293)	1.15 (0.878,1.497)	0.85 (0.636,1.122)	-
Admission, long LoS	0.53 (0.452,0.620)	0.51 (0.374,0.686)	0.66 (0.519,0.848)	0.62 (0.468,0.828)	0.80 (0.492,1.287)
Admission, prolonged LoS	0.86 (0.730,1.003)	0.71 (0.501,0.999)	0.88 (0.630,1.227)	0.57 (0.407,0.785)	0.41 (0.246,0.692)
**to: Death within the first days after admission‡**					
Admission or Discharge	0.93 (0.889,0.978)	0.91 (0.821,1.002)	1.11 (1.009,1.232)	0.95 (0.858,1.042)	1.02 (0.829,1.243)

** Estimated hazard ratios (HRs) with robust 95% confidence intervals (CIs) in clock-reset Cox regression models. The HRs were adjusted for sex, age, care need, and medication-based comorbidity score at admission, and the baseline hazards and OGCM HR were stratified by the relative length of hospital stay (LoS) (defined by the 30%, 60%, and 90% quantiles of each combination of OGCM and index fracture). † HRs were estimated only where at least twenty events were observed in both hospitals with and without OGCM. ‡ The stratified HRs of the time spent in each LoS level were compared with the HRs in a clock-forward model of the time since hospital admission, where "the first days after admission" refers to the median LoS in hospitals with OGCM.*

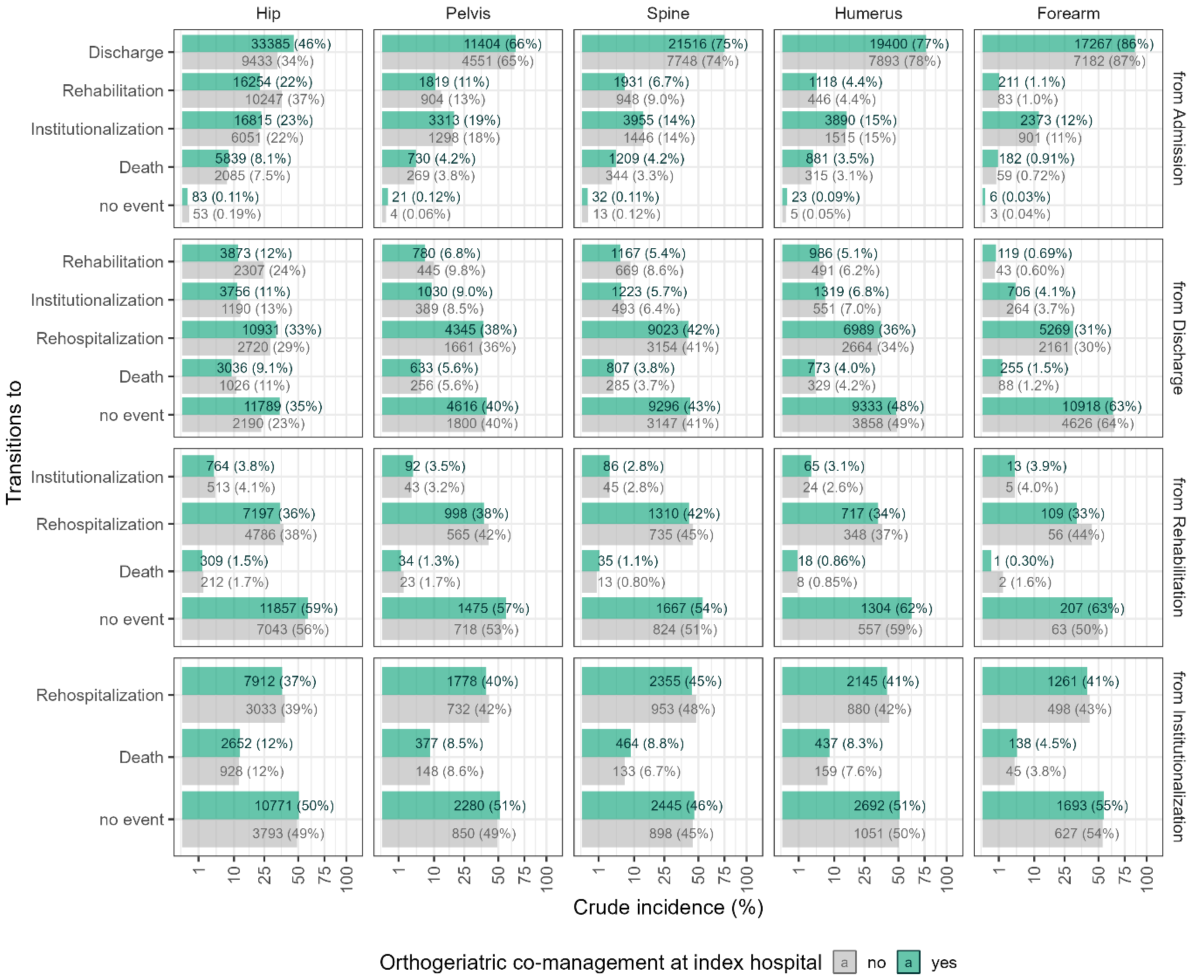

Figure S1: Crude transition incidences as event frequencies ("transition to") with proportions among all patients with the same discharge state ("from"). The incidences are stratified by the type of index fracture and the level of orthogeriatric co-management at the index hospital.

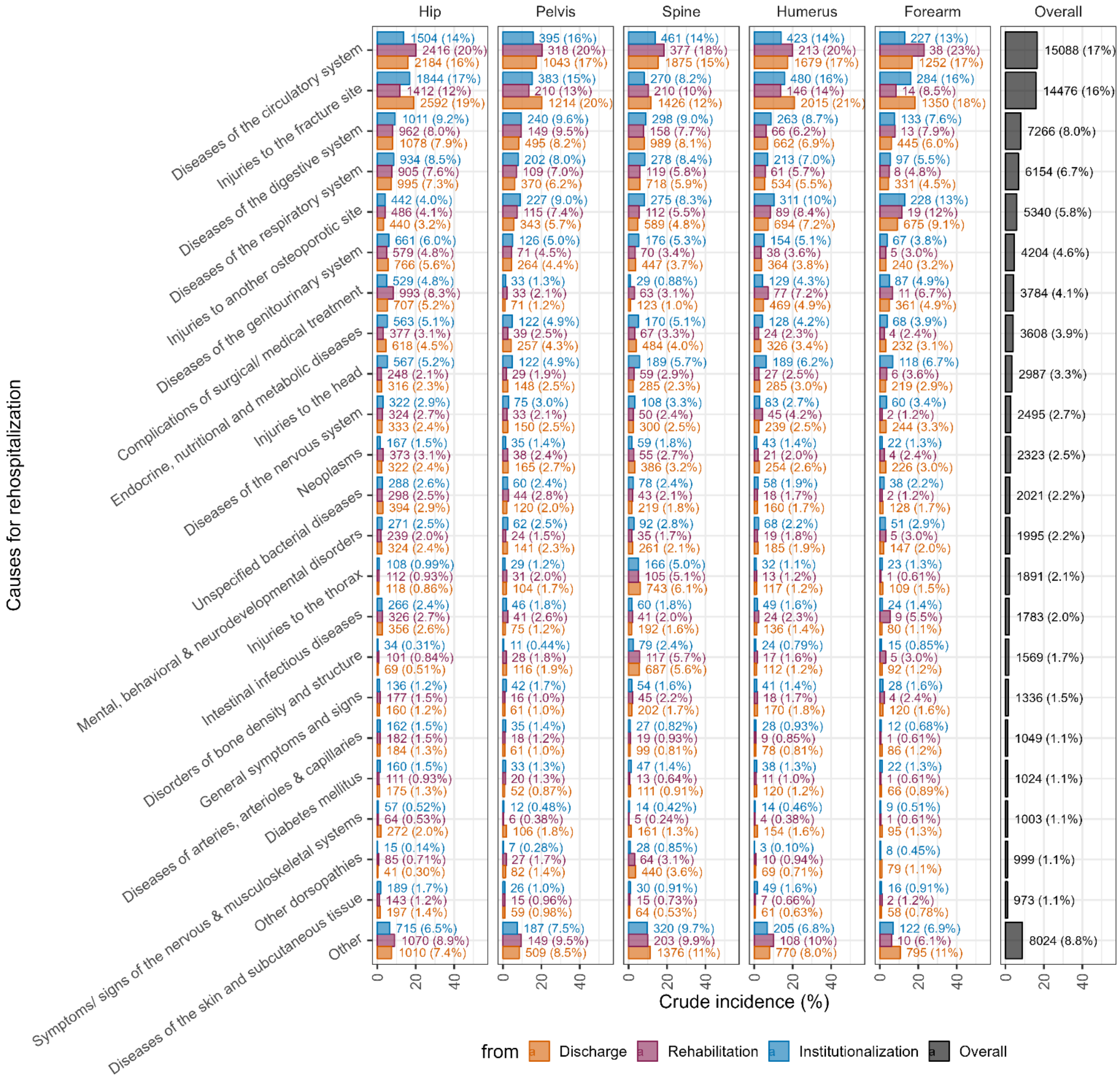

Figure S2: Causes for rehospitalization among all rehospitalized patients. Presented are the frequencies and percentages by type of index fracture and discharge location ("from"). The causes are ordered by their overall frequencies. Causes observed within ≤1% of rehospitalized patients are summarized as "other".

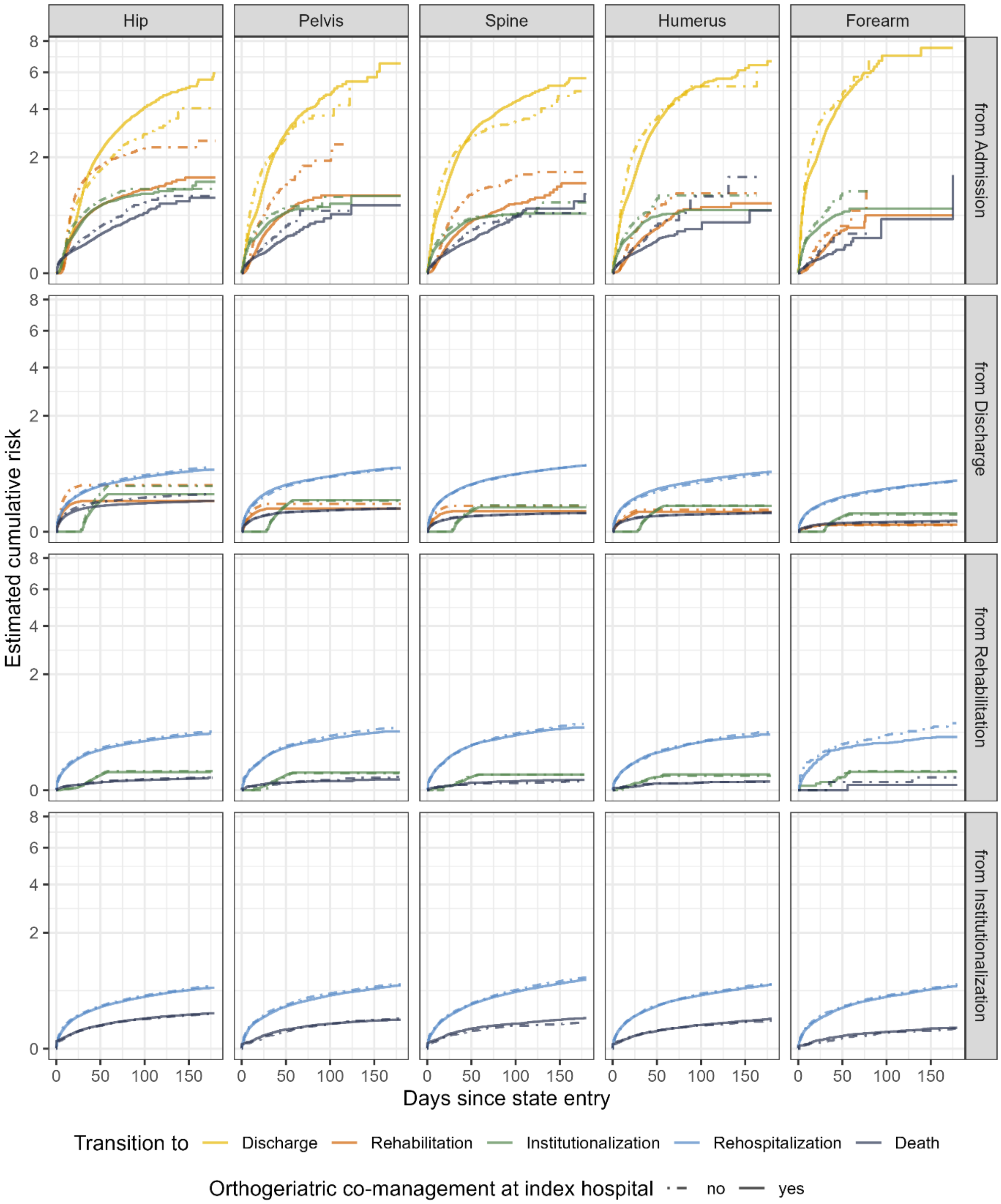

Figure S3: Nelson-Aalen estimator for the cumulative transition hazards (cumulative risk) in the multistate model, stratified by the type of index fracture and the level of orthogeriatric co-management at the index hospital.

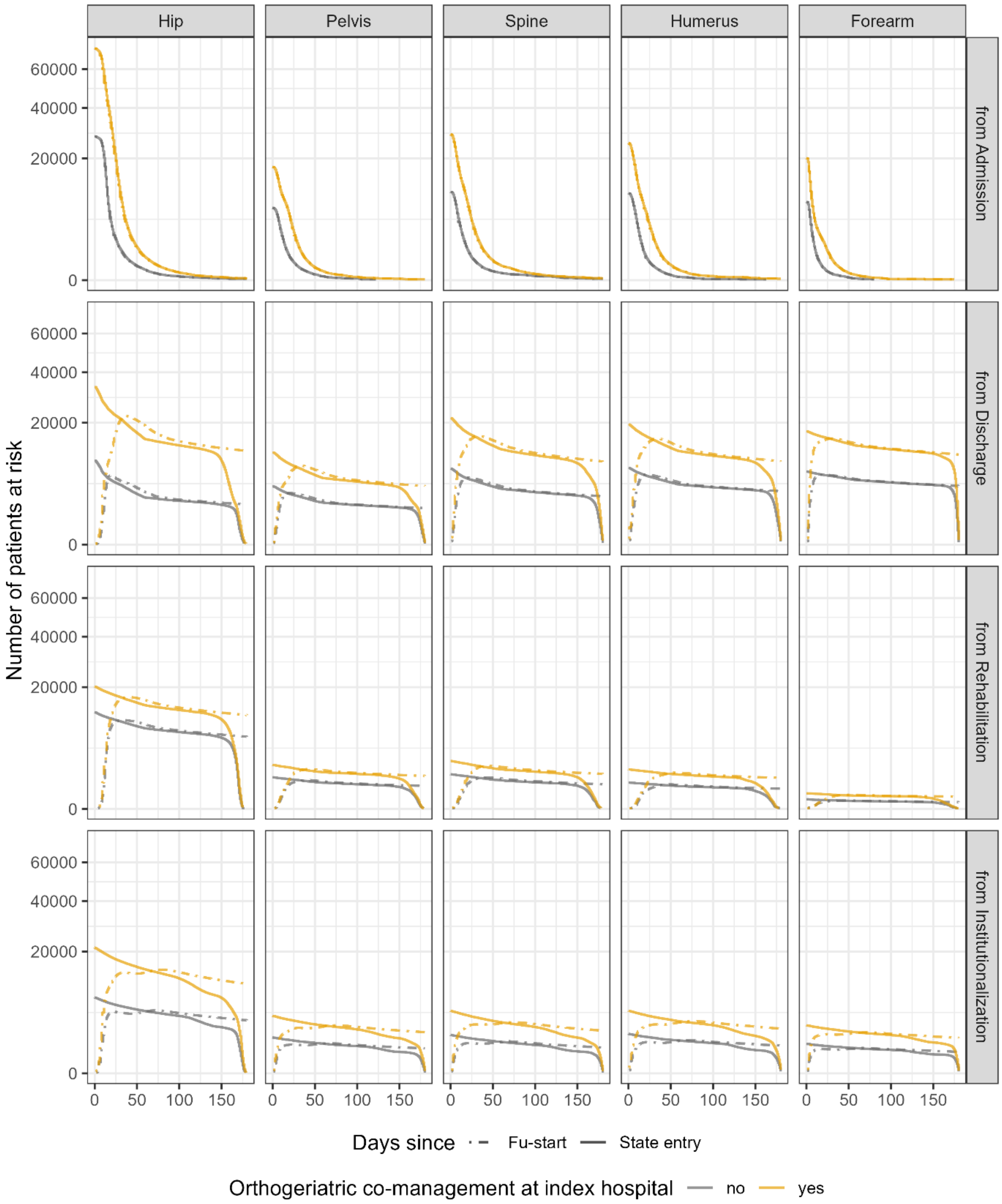

Figure S4: Size of the risk set for transitions in the multistate model on two different time scales (days since follow-up (fu) start or state entry), stratified by the type of index fracture and the level of orthogeriatric co-management at the index hospital.

References

1. Iacobelli S, Carstensen B. Multiple time scales in multi-state models. Stat Med. 2013;32:5315–27.

2. Tutorial in biostatistics: competing risks and multi-state models - Putter - 2007 - Statistics in Medicine - Wiley Online Library. https://onlinelibrary.wiley.com/doi/10.1002/sim.2712. Accessed 4 Mar 2025.

3. Clark DE, Ostrander KR, Cushing BM. A Multistate Model Predicting Mortality, Length of Stay, and Readmission for Surgical Patients. Health Serv Res. 2016;51:1074–94.

4. Kates SL, Behrend C, Mendelson DA, Cram P, Friedman SM. Hospital readmission after hip fracture. Arch Orthop Trauma Surg. 2015;135:329–37.
